# Cerium Nitrate Stiffens *In Vitro* Skin Models and Reduces *Pseudomonas aeruginosa* Pathogenicity and Penetration Through Skin Models

**DOI:** 10.1089/wound.2022.0026

**Published:** 2023-07-27

**Authors:** Shankar J. Evani, Ping Chen, S.L. Rajasekhar Karna, Peter D'Arpa, Kai P. Leung

**Affiliations:** Combat Wound Care Group, United States Army Institute of Surgical Research, Fort Sam Houston, Texas, USA.

**Keywords:** cerium nitrate, burn, *Pseudomonas*, collagen, *ex vivo*-porcine skin, virulence

## Abstract

**Objective:**

Cerium nitrate (CeN) plus silver sulfadiazine (SSD) cream has been used for 40-plus years to manage burns. CeN produces a hardened eschar believed to resist bacterial colonization/infection. To evaluate this potential mechanism, we treated *in vitro* skin models or *Pseudomonas aeruginosa* with CeN and measured mechanical properties of the models and bacterial virulence, respectively.

**Approach:**

We treated three-dimensional-collagen matrix and *ex-vivo*–burned porcine skin with CeN and evaluated stiffness and *P. aeruginosa* penetration. In addition, we treated *P. aeruginosa* with CeN and evaluated the bacteria's motility, skin model penetration, susceptibility to be phagocytized by the human monocytic cell line THP-1, and ability to stimulate this cell line to produce cytokines.

**Results:**

CeN treatment of skin models stiffened them and made them resistant to *P. aeruginosa* penetration. Inversely, CeN treatment of *P. aeruginosa* reduced their motility, penetration through skin models (*ex-vivo*–burned porcine skin), and ability to stimulate cytokine production (tumor necrosis factor-α [TNF-α] and interleukin 8 [IL-8]) by THP-1 cells. In addition, CeN-treated *Pseudomonas* was more readily phagocytized by THP-1 cells. Finally, *P. aeruginosa* inoculated on CeN-treated *ex-vivo*–burned porcine skin was more susceptible to killing by a silver dressing.

**Innovation:**

*In vitro* skin models offer a platform for screening drugs that interfere with bacterial penetration into wounded tissue.

**Conclusion:**

CeN treatment reduced *P. aeruginosa* virulence, altered the mechanical properties of *ex-vivo*–burned porcine skin and collagen matrix, retarded penetration of *P. aeruginosa* through the skin models, and resulted in increased vulnerability of *P. aeruginosa* to killing by antimicrobial wound dressings. These data support the use of CeN in burn management.

**Figure:**
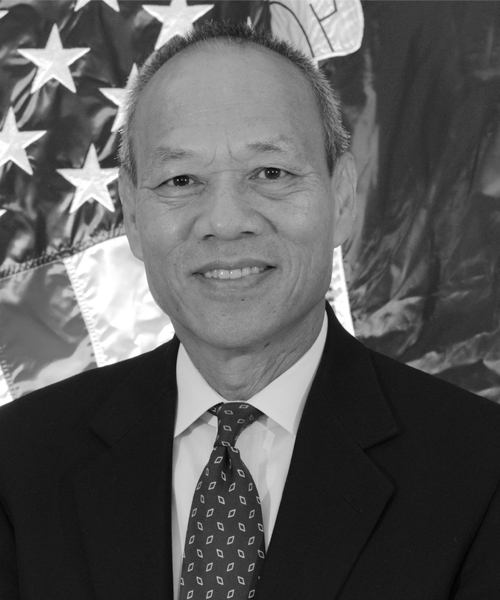
Kai P. Leung, PhD

## INTRODUCTION

Severe burn injuries pose a significant problem worldwide, disproportionately affecting military populations (combat or noncombat operations).^1^ Burns disrupt the skin barrier, local blood supply,^2^ and immune function,^3^ making them susceptible to colonization and infection by endogenous normal skin flora and exogenous bacteria. Infections of burn wounds can delay healing and contribute to hypertrophic scarring, nonhealing wounds, sepsis, multiorgan failure, and mortality.^4^

Infection is the leading cause of mortality responsible for up to 50% of burn related deaths,^5^ and ∼75% of deaths in patients with 40% total body surface area burn.^6^ A frequently encountered pathogen infecting burn wounds is *Pseudomonas aeruginosa*, a highly invasive species.^7,8^ Tribble et al (2016) showed that *P. aeruginosa* is the most common gram-negative bacteria isolated from the combat-related injuries in wounded military personnel in the Afghanistan Operation Theater.^9^

To reduce the risk of burn infection, the standard care includes topical antimicrobials (mafenide acetate, silver sulfadiazine [SSD], chlorhexidine, and other combinatorial drugs) and early excision of the burn eschar and skin autografting. However, eschar excision and grafting requires a sterile operation room, highly skilled surgeons, equipment, and available donor skin or other coverage material and is only practiced under specialized hospital settings^10,11^ that are not available on the battlefield or in resource-limited regions of the world.^12,13^ For such environments, wound care products are needed that can temporize burn wounds, such as by stabilizing the eschar, reducing its toxicity, and mitigating microbial colonization and infection. In hospital settings, one product that has been used for these purposes for 40-plus years is the Flammacerium^®^ cream containing cerium nitrate (CeN, 2.2 wt %) and SSD (1.0 wt %).

CeN has broad spectrum antibacterial activity. Burkes and McCleskey performed a systematic investigation of the bacteriostatic properties of salts of cerium, lanthanum, and thallium against a panel of 39 bacterial species across 16 genera, including gram-positive *Staphylococcus aureus* and gram-negative *P. aeruginosa*. Using radial streak inoculations (eight cultures to each plate) on petri plates of solidified agar containing the specified amount of the salts and basal media, they found that CeN was an effective bacteriostatic agent against the whole spectrum of bacteria. *Pseudomonas* species were the most susceptible genera, with inhibition of growth at concentrations 0.1–0.4 mM of CeN followed by *Escherichia* and *Salmonella* species at 0.5 mM. However, almost twice that concentration is effective on *S. aureus.*^14^

Marone et al evaluated the antimicrobial activity of SSD, alone and in combination with CeN, gentamicin, and amikacin against 130 clinical isolates, including multidrug resistant bacteria such *as P. aeruginosa* or methicillin-resistant *S. aureus* (MRSA). The results showed that the combination of SSD and CeN was as effective as SSD alone.^15,16^

In these early studies CeN has demonstrated broad but greater antimicrobial activity against gram-negative bacteria at concentrations of 10–40 mM, which led to the use of CeN combined with SSD to complement SSD's gram-positive antimicrobial activity.^17,18^ However, subsequent testing of the complementarity of these agents *in vitro* produced conflicting results,^19^ possibly due to cerium precipitating as cerium phosphate or cerium binding to proteins, as well as SSD having poor solubility giving erroneous results in agar disc diffusion tests or broth assays.^17,20,21^ Nonetheless, topical application of CeN-SSD on patient's burns has been associated with decreased bacterial contamination and *P. aeruginosa* infection.^18,19,22–24^

Alongside CeN's inherent antimicrobial activity, in treating human burn wounds CeN in CeN-SSD produces an eschar that adheres well for longer periods to the underlying tissue and may resist bacterial colonization and invasion.^20,22,25^ Thus, CeN's effects both on the eschar and bacteria may mitigate bacterial colonization and infection of burn wounds.^26,27^ However, little experimental evidence for these mechanisms is available. In this study, we independently treated *P. aeruginosa* and tissue models (three-dimensional [3D]-collagen matrix and *ex-vivo*–burned porcine skin).

**Figure 1.**
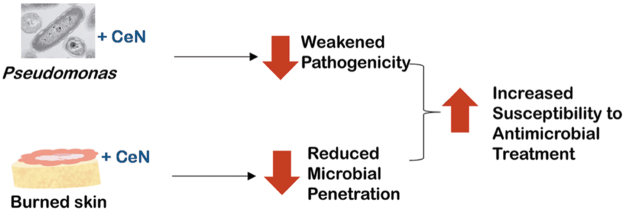
Diagram: Summarizing potential effects of CeN on *Pseudomonas aeruginosa* and on burned skin model leading to reduced virulence, reduced skin penetration, and increased vulnerability to antimicrobial treatment. CeN, cerium nitrate.

After treating the *in vitro* models with CeN, we evaluated their stiffness of *ex-vivo*–burned porcine skin. In addition, after treating the bacteria, we evaluated its survival, motility, and ability to be phagocytized by the human monocytic cell line THP-1 and to stimulate this cell line to produce pro-inflammatory cytokines. Furthermore, the *ex-vivo*–burned porcine skin treated with CeN was tested for *P. aeruginosa* penetration and the sensitivity of bacteria inoculated on it to be killed by a silver dressing. Our results show that CeN acted on *P. aeruginosa* and burn tissue by stiffening the skin models and by affecting the motility of *P. aeruginosa*, resulting in lesser penetration through the skin models (Fig. 1). Furthermore, CeN increased the susceptibility of *P. aeruginosa* to phagocytosis by the THP-1 cells and to the killing by antimicrobial agents such as SSD or Ag.

## CLINICAL PROBLEM ADDRESSED

CeN-SSD treatment enhances the adherence of the eschar to underlying tissue while producing a dried wound that resists infection and facilitates postponement of surgical eschar excision and grafting. Additional benefits reported for CeN-SSD treatment are fewer dressing changes, less soak-through of exudate, less labor-intensive care, and greater comfort for patients.^20,24,28,29^ In this study, in *in vitro* skin models we show that CeN stiffens burn tissue and makes it more resistant to *Pseudomonas* penetration. In addition, CeN treated-*P. aeruginosa* was less virulent and more susceptible to antimicrobials. This evidence supports the use of CeN in the management of burn wounds.

## MATERIALS AND METHODS

### Bacterial strains, cell line, and culture conditions

Three *P. aeruginosa* strains were used in this study: strain PAO1 (ATCC, Manassas, VA), PAO1 transformed with a red-fluorescent expression plasmid pMT RFP (provided by Dr. Seok Jong Hong, Northwestern Medical School), and a clinical strain 1244.^30^ All strains were grown in tryptic soy broth (TSB) and grown overnight or to mid-log growth phase (OD_600nm_ = ∼0.1–0.4).

For phagocytosis and inflammatory response assays, THP-1 cell suspensions (ATCC) were used. The cells were propagated for four generations before storing in a −150°C freezer. For each experiment, THP-1 cells were freshly thawed, washed once with Hank's balanced salt solution (with phenol red but without Ca^2+^ and Mg^2+^; Hank's balanced salt solution [HBSS]; Gibco-Life Technologies, Carlsbad, CA), and propagated in media containing 45% RPMI 1640 (ATCC), 45% HBSS, and 10% fetal bovine serum (FBS; Gibco-Life Technologies) for a maximum five passages in TC- Flask T75 Standard, Vented Cap (Ref. 83.3911.002; SARSTEDT). The THP-1 cells were grown to a density of ∼10^6^ per mL at 5% CO_2_ and 37°C, and the treatment assays were performed in the 5 mL polypropylene round-bottom tubes (Ref. 352063; Corning Sciences). CeN [Cerium(III) nitrate hexahydrate, 99.5%, CAS No. 10294-41-4] was obtained from Arcos.

### Preparation of 3D-collagen gels and measurements of mechanical/physical changes

Collagen gels were prepared as per the manufacturer's instructions with the pregelation procedure carried out on ice with cold-incubated pipette tips. Briefly, collagen (Collagen I heavy chain [HC], rat tail −0.02 N in Acetic Acid; BD Biosciences, Bedford, MA) was mixed with HBSS (10% v/v without Ca^++^ or Mg^++^), neutralized with sodium bicarbonate, and equilibrated to 5 mg/mL collagen in HBSS. Gelation was carried out at room temperature for 30 min, and the gels were further incubated at 37°C for a minimum of 1 h.

**Figure 2.**
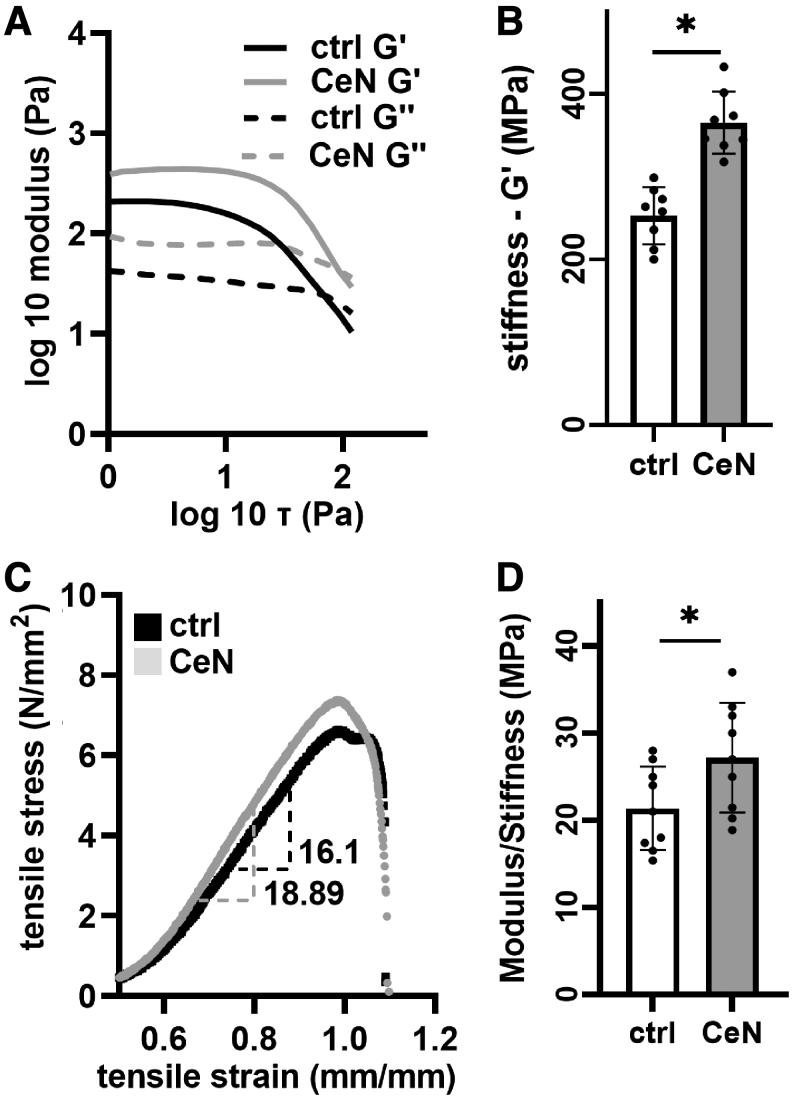
CeN altered mechanical properties of 3D-collagen gel and *ex-vivo*–burned porcine skin: Collagen gels were treated with 1 mM CeN or water control (ctrl) for 30 min followed by a 48-h incubation at 37°C without treatment. Viscoelastic properties were measured by performing amplitude sweep at 1 Hz using a rheometer. **(A)** Representative flow curves for collagen gel. **(B)** Calculated stiffness (G′) for collagen gel. In a separate experiment, burned porcine skins were treated as for the gel but with 40 mM CeN and measured for **(C)** Tensile stress versus tensile strain curves and **(D)** uniaxial tensile modulus/stiffness. Data points represent mean ± SD from three experiments done in triplicate. *Significant difference (*p* ≤ 0.05; *t*-test) between groups. 3D, three-dimensional; ctrl, control; SD, standard deviation.

For measuring the physical property change, 280 μL collagen gels were incubated with 280 μL of 0 or 1 mM CeN for 30 min. After incubation, CeN solution was removed, and the gels were blot dried and incubated for 2 days at 37°C before the measurements. Oscillatory amplitude sweep with 1 to 100 Pa at an angular frequency of 1 Hz, as described by Evani et al,^31^ was used to calculate viscoelastic properties of the gels using the HAAKE MARS modular advanced rheometer (ThermoFisher Scientific, Inc.) equipped with parallel plate-PP20. Storage modulus in linear viscoelastic range was measured as an indicator stiffness (Fig. 2).

### *P. aeruginosa* PAO1 penetration of 3D-collagen gel

To visualize *P. aeruginosa* PAO1 penetration, green fluorescent-labeled collagen (Collagen Type I-FITC Conjugate from bovine skin, ∼1 mg/mL solution, Cat. No. C4361; Sigma, St. Louis, MO) was used. Collagen gels were prepared as described above. Briefly, green fluorescent-labeled collagen was added at 10% v/v before neutralization. Next, 120 μL of this collagen was dispensed into the wells of eight-well cover-glass chamber slides (LabTek-1; ThermoFisher Scientific Inc.). The preformed gels were then incubated with 120 μL of CeN solution (0 or 1 mM) for 30 min. The gels were removed from the CeN solution and blot dried before incubation for 2 days at 37°C in humidified petri dishes.

**Figure 3.**
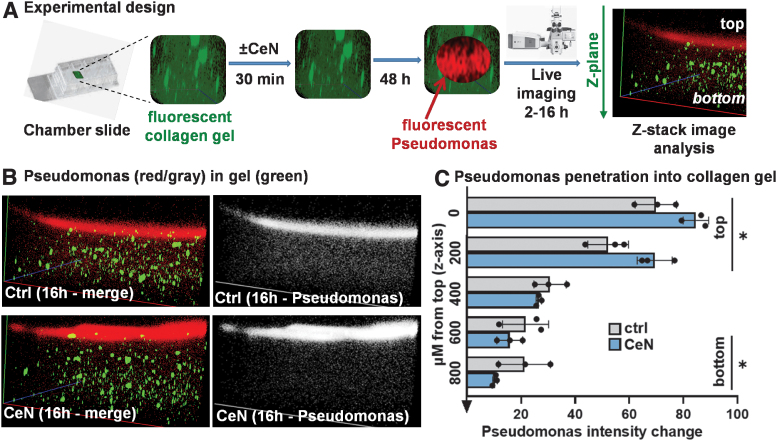
CeN attenuated *Pseudomonas aeruginosa* PAO1 penetration of 3D-collagen gels: **(A)** Experimental design. Collagen gel (*green fluorescent* labeled) was treated with 0 (ctrl) or 1 mM CeN (CeN) for 30 min, blotted dry, and incubated at 37°C for 48 h. Then ∼10^7^ CFU *P. aeruginosa* PAO1 (*red fluorescent* labeled) in 10 μL saline was pipetted on top. After 2 or 16 h, fluorescent images were taken down the z-stack: *P. aeruginosa* (*red*) and gel (*green*). **(B)** Representative 3D images of *green-fluorescent* gel with *red-fluorescent P. aeruginosa* or *gray scale image* of *red-fluorescent P. aeruginosa* in control and CeN-treated collagen. **(C)** The *P. aeruginosa* red fluorescence intensity at 16 h in *z*-planes of the gel (as a ratio of the fluorescence at 2 h); *p ≤ 0.05, two-sided Mann–Whitney U test. CFU, colony-forming unit.

Red-fluorescent-tagged PAO1 (mid-log-phase, ∼10^7^ colony-forming unit [CFU] in 10 μL) was added to the top of the gel and their penetration into it was measured after 2 and 16 h by taking Z-stack images (Zeiss 710-live imaging confocal microscope, Thornwood, NY) at a magnification of 10 × and at the same laser power, gain, and digital offset (Fig. 3A). The images were analyzed over time for changes in red fluorescence intensity from the top to the bottom of the Z stack (Carl-Zeiss Zen software). The fluorescence at 16 h as a ratio of the 2 h fluorescence was plotted as a measure of bacterial penetration.

### Mechanical properties of *ex-vivo*–burned porcine skin

Porcine skin (Description 59490-0 PG Skin; Pel-Freez Biologicals, Rogers, AR) stored at −80°C was thawed to room temperature and cut into dumbbell-shaped pieces. The skin pieces were placed in a 50 mL conical tube containing betadine solution (NDC 67618-150-09; Purdue Products, Stamford, CT) and were slowly mixed at 20 RPM (revolutions per minute) on a tube rotator for 10 min. After treatment with betadine, the skin pieces were washed 3 × in phosphate-buffered saline (PBS, 1 × ) by mixing at 80 RPM for 10 min. These skin pieces were pat dried with sterile gauze and then burned for 13 s at ∼100°C using an in-house burn device.

Burned skin pieces were incubated with either CeN (0 or 40 mM) or CeN (40 mM) + SSD (30 mM) in 50 mL tubes and incubated with slow rotation (20 RPM) for 30 min at room temperature on a tube rotator. The skin pieces were removed from either CeN or CeN+SSD solutions, blot dried, and incubated for 2 days at 37°C in humidified petri dishes (described in *P. aeruginosa* 1244 Penetration of *Ex-Vivo*–Burned Porcine Skin section), before performing the uniaxial tensile strength test (ElectroPuls™ E3000 instrument employing Dynacell™ load cell; Instron, Norwood, MA). Stiffness of the skin was assessed as the ratio of tensile stress to tensile strain from the test outputs from the instrument software.

### *P. aeruginosa* 1244 penetration of *ex-vivo*–burned porcine skin

#### Viable counts from filters

**Figure 4.**
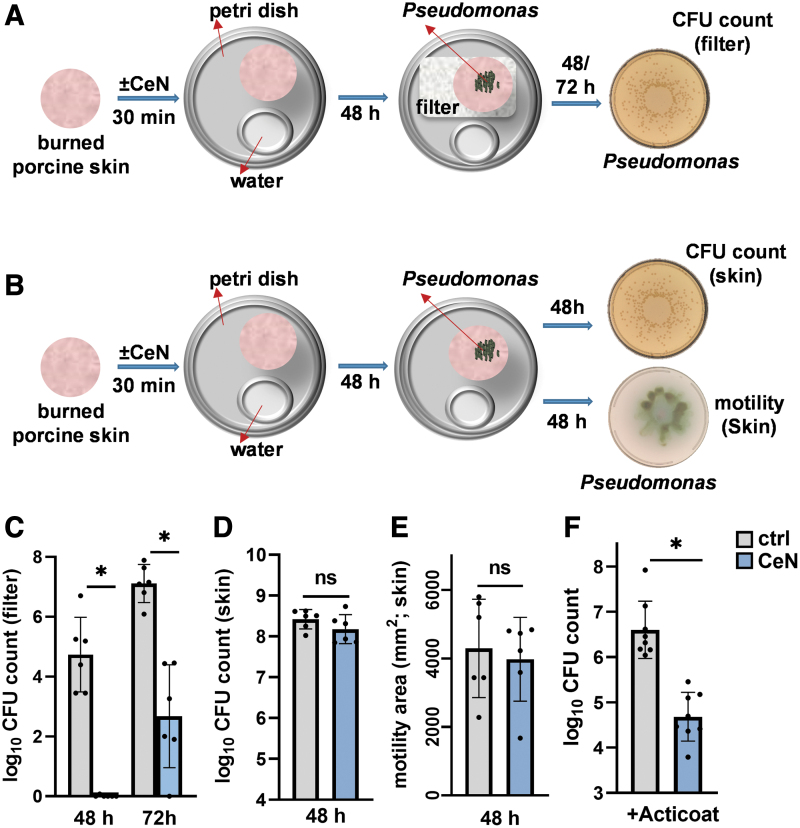
CeN attenuated *Pseudomonas aeruginosa* penetration through burn porcine skin samples **(A–D)** and increases bacterial susceptibility to killing by silver dressing **(E)**: **(A)** Experimental design. Porcine skins were cut into discs and burned for 13 s at 100°C and then treated with 40 mM CeN (CeN) or water (ctrl) for 30 min before incubating at 37°C for 48 h before being placed on *top* of **(A)** 0.2 μm filters or **(B)** with no filters. Approximately 10^6^ CFU *P. aeruginosa* 1244 in 10 μL saline was then added on *top* of the skin discs and incubated for 48 or 72 h before homogenizing the filters or skin to extract *P. aeruginosa* for quantification of viability and motility as described in *P. aeruginosa* 1244 Penetration of *Ex-Vivo*–Burned Porcine Skin section. **(C)** *P. aeruginosa* CFU counts from filters and **(D)** skin discs. **(E)** Motility of *P. aeruginosa* recovered from skin discs. **(F)** *P. aeruginosa* CFU counts from CeN-treated or untreated burn porcine skin discs inoculated with *P. aeruginosa* and treated with moistened Acticoat silver dressing. Data points in the graphs represent mean ± SD obtained from three experiments done in triplicate. *Significant difference between groups (*p* ≤ 0.05; ANOVA/*t*-test). ANOVA, analysis of variance.

Porcine skin was cut into discs (1.1 cm diameter) and processed as described above (Mechanical Properties of *Ex-Vivo*–Burned Porcine Skin section). These skin discs were then placed on top of 0.2 μM filters (Whatman; Sigma-Aldrich). Mid-log-phase *P. aeruginosa*-1244 (∼10^6^ CFU in 10 μL) was pipetted onto the surface of each burned skin. After 2 or 3 days of incubation at 37°C in humidified petri dishes (sterile water in a small container to maintain moisture inside the petri dish, Fig. 4A), the 0.2 μM filters were separated from the replicate skin discs and homogenized in 1 mL PBS in MagNA Lyser Beads tubes (Roche) using FastPrep^®^-24 Tissue Homogenizer (MP Biomedicals, LLC. Santa Ana, CA).

The homogenized samples were plated on *P. aeruginosa* isolation agar (Cat. No. P0144; Teknova, Hollister, CA) and incubated at 37°C for ∼18 h. The viable counts (CFUs) of the 0.2 μM filters were determined using an automated colony counter (ProtoCOL 3 instrument form SYNBIOSIS, Frederick, MD).

#### Viable counts from skin

Porcine skin was cut into discs (1.1 cm diameter) and processed as described above (Mechanical Properties of *Ex-Vivo*–Burned Porcine Skin section). Mid-log-phase *P. aeruginosa*-1244 (∼10^6^ CFU in 10 μL) was pipetted onto the surface of each burned skin. After 2 days of incubation at 37°C in humidified petri dishes (described above), the replicate skin discs were removed from the petri dishes and were separately homogenized in 1 mL PBS in MagNA Lyser Beads tubes (Roche) using a FastPrep-24 Tissue Homogenizer (MP Biomedicals, LLC). The homogenized samples were plated on *P. aeruginosa* isolation agar and incubated at 37°C for 18 h. The viable counts of skin discs were determined using an automated colony counter as described above.

### Antimicrobial activity of Acticoat against *P. aeruginosa* 1244 inoculated on *ex-vivo*–burned porcine skin ± CeN treatment

As described above, the skins were cut into discs, betadine treated, washed with PBS, tap dried with sterile gauze, burned, treated with 40 mM CeN, and incubated for 2 days at 37°C. The burned-skin discs were then inoculated with ∼10^6^ CFU of *P. aeruginosa*-1244 per skin disc and 4 h later were overlaid with Acticoat dressing (Smith & Nephew) that was wetted with sterile H_2_O. After incubation for 2 days at 37°C, Acticoat was removed and the burned-skin discs were homogenized in 1 mL PBS in MagNA Lyser Beads tubes (Roche) using a FastPrep-24 Tissue Homogenizer (MP Biomedicals, LLC). The homogenized samples were plated on *P. aeruginosa* isolation agar, incubated at 37°C for 18 h, and the viable CFU of skin discs was determined using an automated colony (as described in above section).

### *P. aeruginosa* PAO1 viable counts, motility (swarming and twitching), inflammatory-stimulation activity, and susceptibility to phagocytosis by THP-1 cells

#### Viable counts

Overnight *P. aeruginosa* PAO1 culture was serially diluted and adjusted 10^6^ CFU in 10 μL with the sterile saline solution (0.9% w/v, pH7.0) and was mixed with 990 μL of saline solution containing different concentrations of CeN (0, 0.1, 1, and 10 mM). After incubation at 25°C for 24 h with shaking, these solutions were serially diluted and plated on *P. aeruginosa isolation* agar, incubated at 37°C for 18 h. The viable cells (CFU) recovered from the CeN treatment were determined using an automated colony counter (as described in above *P. aeruginosa* 1244 Penetration of *Ex-Vivo*–Burned Porcine Skin section).

#### Swarming motility

Assays were performed on plates containing 0.5% w/v Bacto agar (Difco), 8 g of nutrient broth (Oxoid) l-1, and 0.5% w/v D-glucose as previously described.^32,33^ Swarming motility was performed on *P. aeruginosa* PAO1 cells that were treated with different CeN concentrations (or) bacteria recovered from the CeN treated and untreated skins and normalized to ∼10^6^ CFU/mL. Five microliters of culture was spotted in four replicates. Swarming was observed after 18 h of incubation at 37°C. The zone of the swarming motility area was determined by measuring the mean diameter of the motility circle.

#### Twitching motility

Assays were performed on plates containing 10 g/L tryptone, 5 g/L yeast extract, and 0.5% w/v D-glucose solidified with 1.0% (w/v) Difco granulated agar as previously described.^34^ *P. aeruginosa* PAO1 cells were treated with different concentrations of CeN and normalized to 10^6^ CFU/mL. The cells were pelleted and resuspended in 50 μL of the LB media. A sharp toothpick, which has been immersed in the above bacterial suspension, was used to stab through the agar to the bottom of the 1-day-old plates. The plates were then incubated at 25°C for 24 h, and the zone of twitching motility area at the agar/petri dish interface was determined by measuring the mean diameter of the motility circle.

#### Inflammatory-stimulation activity

*P. aeruginosa* PAO1 cells were treated with different concentrations of CeN, spun down, and heat inactivated (65°C for 1 h). Dead bacteria (confirmed by plating) were added (10^6^) to THP-1 cells (5:1 ratio bacteria to THP-1 cells) in 500 μL and incubated for 1 h at 37°C. Supernatants were then assayed for tumor necrosis factor-α (TNF-α) and interleukin 8 (IL-8; enzyme-linked immunosorbent assay [ELISA] kit; R&D Systems, Minneapolis, MN) on a DSX automated ELISA machine (Dynex Technologies) as described previously.^35^

In a separate experiment, for the mock group, THP-1 cells (10^6^ THP-1 cells; 500 μL total volume) were incubated with 0 mM CeN (control [ctrl]) or 0.1 mM CeN. For the experimental group, the THP-1 cells (10^6^ THP-1 cells; 500 μL total volume) were incubated with lipopolysaccharide (LPS; Cat. No. L9143, *P. aeruginosa*; Sigma), either untreated or pretreated with 0.1 mM CeN for 30 min. After incubation for 1 h at 37°C, supernatants were collected and then quantified for TNF-α and IL-8 as described above.

#### Phagocytosis assay

*P. aeruginosa* PAO1 cells were treated with CeN (or saline control) and were stained with live stain (green; live/dead bacterial staining kit; Invitrogen). In another experiment, the CeN-treated bacteria were heat inactivated at 65°C for 45 min and then stained with dead cell stain (red; live/dead bacterial staining kit; Invitrogen). The stained bacteria were added to 10^6^ THP-1 cells (5:1 ratio of normalized live or heat killed bacteria to THP-1 cells in a total volume of 500 μL) for 1 h incubation at 37°C. Before the THP-1 cells were spun down, they were stained with corresponding Cytotox-red (or) green stain (Incucyte, Cat. No. 4632 (red); 4633 (green); Essen BioScience, Inc., Ann Arbor, MI) and assessed for fluorescence from bacterial phagocytosis.

Bacterial phagocytosis was assayed as the percentage of cells containing green fluorescent (live) or red fluorescent (dead) bacteria and the mean fluorescence intensity (MFI) of the phagocytized bacteria using the Attune NxT flow cytometer (Invitrogen-Life Technologies, Carlsbad, CA). Viable THP-1 cells were gated on forward scatter and negative events from corresponding Cytotox-fluorescence (dead THP-1 cells). Unstained and compensation controls were used to gate the events positive for phagocytosis.

### Statistical analyses

Experiments were performed in duplicate or triplicate, and each experiment was repeated a minimum of three times on different days. All statistical analyses were performed in GraphPad Prism 9 (GraphPad Software, San Diego, CA). Values are presented as mean with standard deviation, unless otherwise stated. Significant differences between two groups were established by *t*-test or two-tailed Mann–Whitney test for nonparametric data. For testing significant differences among multiple groups, one-way analysis of variance with Holm-Šídák's multiple comparisons test was used.

Electronic laboratory notebook was not used.

## RESULTS

### Stiffness of both 3D collagen and *ex-vivo*–burned porcine skin was increased by CeN

CeN treatment (1 mM) of collagen gel increased stiffness detected as storage modulus (G′, kPa) (Fig. 2A, B). Similarly, burned porcine skins treated with 40 mM CeN demonstrated increased modulus stiffness (Fig. 2C, representative tensile stress vs. strain curves) and stiffness (Fig. 2D and Supplementary Fig. S1). Burned porcine skin either treated with CeN (40 mM) alone or combination of CeN (40 mM) and SSD (30 mM) showed comparable increases of stiffness (Supplementary Fig. S2).

### Penetration of *P. aeruginosa* through CeN-treated 3D-collagen gel or *ex-vivo*–burned porcine skin was retarded

As shown in the experimental design (Fig. 3A), red-fluorescent-labeled *P. aeruginosa* PAO1 was pipetted on top of collagen gels that had been treated with or without 1 mM CeN, and 2–16 h later the red fluorescence intensity of *P. aeruginosa* in layers of the gel was measured. In the gel that was pretreated with CeN, *P. aeruginosa* PAO1 was increased over time in upper layers and decreased in lower layers (Fig. 3B, C).

Similarly, we assessed penetration of *P. aeruginosa* strain 1244 through CeN-treated *ex-vivo*–burned porcine skin. *P. aeruginosa* 1244 was pipetted onto the surface of the CeN-treated skin (schematic in Fig. 4A, B). After 2 or 3 days of incubation at 37°C, the number of *P. aeruginosa* recovered from filters underneath the skin was assessed (Fig. 4A). After 48 h, ∼10^6^ bacteria were recovered under the untreated skin while under the CeN-treated skin no bacteria were recovered (Fig. 4C). After 72 h postinoculation, ∼10^2^ CFU was recovered from the filters under the CeN-treated skin, while ∼10^7^ CFU was recovered from the filters under the untreated skin.

### *P. aeruginosa* 1244 recovered from *ex-vivo*–burned porcine skin discs treated with or without CeN had similar viable counts and motility

*P. aeruginosa* 1244 was pipetted onto the surface of the CeN-treated skin. Bacteria were recovered after 2 days of incubation at 37°C for determination of viable counts (Fig. 4B). The number of viable bacteria recovered from the treated and untreated skin discs was not significantly different (Fig. 4D).

For swarming motility assay, bacteria recovered from the CeN-treated and untreated skins as described above were plated on soft agar. After 18 h incubation at 37°C, the area of bacterial spread around the spot of inoculation was measured. There was no significant difference in the area of the bacteria recovered from the treated and untreated skin discs (Fig. 4E).

### Greater killing by silver dressing of *P. aeruginosa* inoculated on *ex-vivo*–burned porcine skin treated with CeN

*Ex-vivo*–burned porcine skin discs were untreated or treated with CeN (40 mM), inoculated with *P. aeruginosa* 1244, and 4 h later was overlaid with silver dressing. The skins were then homogenized to extract bacteria for determining viable counts. Viability of the bacteria was significantly lower (∼2-logs) in the CeN-treated porcine skin overlaid with silver dressing than the untreated porcine skin overlaid with silver dressing (Fig. 4F).

### Viable counts, motility, and penetration through skin models were reduced by treatment of *P. aeruginosa* with CeN

**Figure 5.**
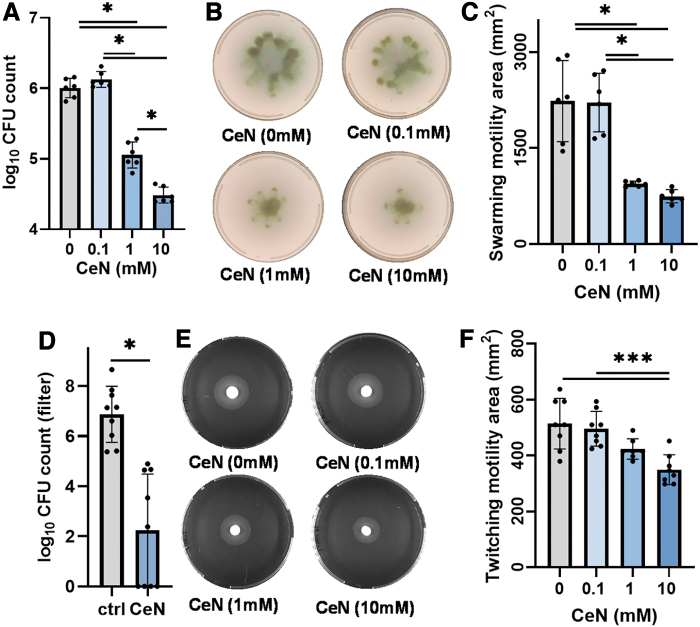
CeN reduced *Pseudomonas aeruginosa* viable counts, motility (twitching and swarming), and penetration: *P. aeruginosa* PAO1 was treated with 0, 0.1, 1, and 10 mM CeN for 24 h and then assayed for viability and motility. **(A)** represents the viable counts (CFUs) of *P. aeruginosa* treated with CeN for 24 h which were enumerated as mentioned in *P. aeruginosa* PAO1 Viable Counts, Motility (Swarming and Twitching), Inflammatory-Stimulation Activity, and Susceptibility to Phagocytosis by THP-1 Cells section. **(B, C)** Swarming motility and **(E, F)** Twitching motility on soft agar plates with area of bacterial spread quantified by image analysis. **(D)** The viable counts of the bacteria recovered from filters underneath *P. aeruginosa* 1244-inoculated burned porcine skin which represented bacterial penetration (as described in *P. aeruginosa* 1244 Penetration of *Ex-Vivo*–Burned Porcine Skin section). Data points represent mean ± SD obtained from three experiments done in triplicate. *^,^***Significant difference (*p* ≤ 0.05; ANOVA/*t*-test).

In the studies above, the collagen gel and the porcine skin were treated with CeN. In this study, *P. aeruginosa* PAO1 was treated directly with different concentrations of CeN for 24 h at 25°C. As shown in Fig. 5A, the viability reduction was concentration dependent, with 10 mM CeN reducing viability by 1.5 logs, while 0.1 mM CeN was not bactericidal compared to untreated control. In addition, CeN dose dependently killed a variety of microbes known to colonize wounds, including *P. aeruginosa*, *Acinetobacter baumannii*, *Klebsiella pneumoniae*, *Enterococcus faecalis*, *S. aureus*, and *MRSA* (Supplementary Fig. S3).

In addition, we measured the swarming motility of CeN-treated *P. aeruginosa* PAO1. The bacteria treated with a range of CeN concentrations were plated on soft agar, and after 18 h incubation at 37°C the area of spread of the bacteria around the spot of inoculation was measured. One to 10 mM but not 0.1 mM CeN reduced swarming motility (Fig. 5B, C). The twitching motility was also measured for *P. aeruginosa* PAO1 treated with series of CeN concentrations. The bacteria were stabbed into the agar, and after 24 h incubation at 37°C, the area of spread of the bacteria around the stab was measured. Compared to 0 and 1 mM CeN, 10 mM CeN showed a significant reduced twitching motility (Fig. 5E, F).

Furthermore, we assessed the ability of CeN-treated *P. aeruginosa* 1244 to penetrate *ex-vivo*–burned porcine skin by determining the number of viable *Pseudomonas* recovered from filters underneath the skin discs. Recovery of the bacteria treated with 10 mM from the filters was reduced by 1.5 logs (Fig. 5D).

### CeN-treated *P. aeruginosa* was more susceptible to phagocytosis by THP-1 cells

**Figure 6.**
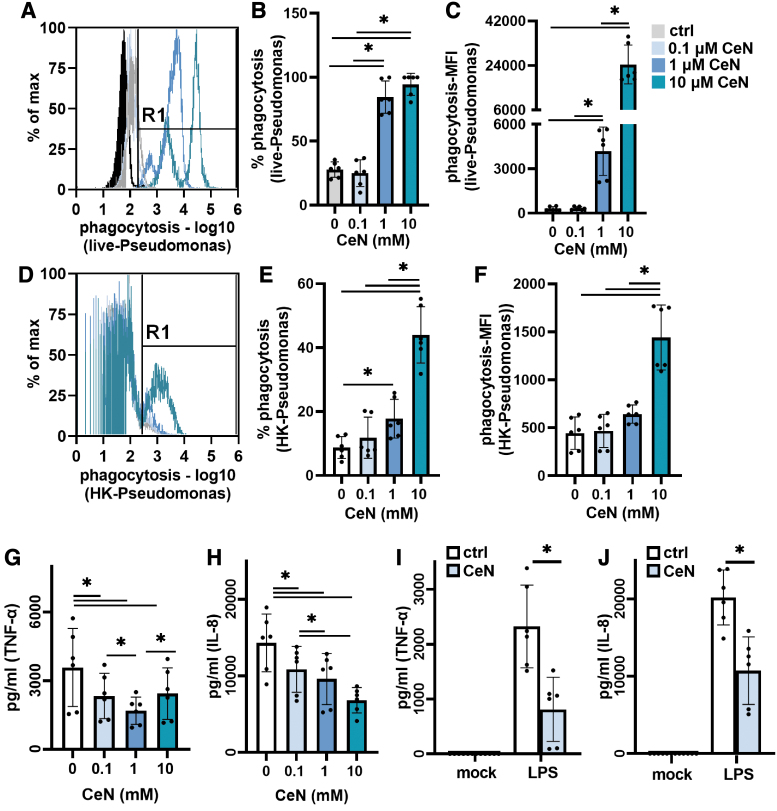
*Pseudomonas aeruginosa* PAO1 treated with CeN was phagocytized more by THP-1 cells and less stimulatory to THP-1 cytokine production: **(A–C)** *P. aeruginosa* PAO1 was incubated with CeN in saline for 24 h. Live bacteria were stained with live bacterial stain (*green*) and incubated with THP-1 cells (5:1) for 1 h and analyzed for *green fluorescence* (live bacteria) from phagocytosis. **(A)** Representative histogram, **(B)** percent of viable THP-1 cells positive for *Pseudomonas green fluorescence*, **(C)** MFI of *Pseudomonas*. **(D–F)** *P. aeruginosa* PAO1 was incubated with CeN in saline for 24 h, heat killed, and stained with dead bacterial stain (*red*) and incubated with THP-1 cells (5:1) for 1 h and analyzed for *red fluorescence* (dead bacteria) from phagocytosis. **(D)** Representative histogram, **(E)** percent of viable THP-1 cells positive for *Pseudomonas red fluorescence*, **(F)** MFI of *Pseudomonas*. **(G, H)** *P. aeruginosa* treated with CeN, heat killed, and incubated with THP-1 cells (5:1) for 1 h before collecting supernatants and assaying for **(G)** TNF-α and **(H)** IL-8 using ELISA. **(I, J)** In another experiment, LPS from *P. aeruginosa* (1 μg/mL) was treated with 0.1 mM CeN for 30 min and then incubated with THP-1 cells. In addition THP-1 cells were treated with 0 or 0.1 mM CeN (mock treatment, *P. aeruginosa* PAO1 Viable Counts, Motility (Swarming and Twitching), Inflammatory-Stimulation Activity, and Susceptibility to Phagocytosis by THP-1 Cells section) for 1 h when supernatants were assayed for **(I)** TNF-α and **(J)** IL-8 using ELISA. Data points are the mean ± SD from three experiments done in duplicate. *Significant difference (*p* ≤ 0.05; ANOVA/*t*-test). ELISA, enzyme-linked immunosorbent assay; IL-8, interleukin 8; LPS, lipopolysaccharide; MFI, mean fluorescence intensity; TNF-α, tumor necrosis factor-α.

To assess CeN treatment of viable *P. aeruginosa* PAO1 on phagocytosis, live CeN-treated *P. aeruginosa* (stained green) was added to THP-1 cells. After a 1-h incubation at 37°C, the percentage of viable THP-1 cells containing live green-fluorescent *P. aeruginosa* was determined by flow cytometry. The bacteria treated with 1 and 10 mM CeN were phagocytized to a significantly greater extent than the untreated bacteria (Fig. 6A–C). In addition, the *P. aeruginosa* treated with 1 and 10 mM CeN had a greater mean green-fluorescent intensity per THP-1 cell.

To assess CeN treatment of *P. aeruginosa* PAO1 on phagocytosis of nonviable *P. aeruginosa*, CeN-treated *P. aeruginosa* was heat killed (stained-red) before incubation with THP-1 cells. After a 1-h incubation at 37°C, the percentage of viable THP-1 cells containing dead red-fluorescent *P. aeruginosa* was determined by flow cytometry. The bacteria treated with 1 and 10 mM CeN that were heat killed were phagocytized to a significantly greater extent than the untreated bacteria (Fig. 6D–F). In addition, the *P. aeruginosa* treated with 10 mM CeN had a greater mean red-fluorescent intensity per THP-1 cell. In addition, in our experimental conditions, the THP-1 cells treated with CeN showed no significant changes in phagocytosis (Supplementary Fig. S4).

### Inflammatory stimulatory activity of *P. aeruginosa* or LPS treated with CeN was reduced

#### Inflammatory activity of CeN-treated *P. aeruginosa*

*P. aeruginosa* PAO1 was treated with CeN and then heat-killed because the live bacteria induced significant THP-1 cell death. The bacteria treated with CeN (0.1, 1, and 10 mM) elicited significantly less TNF-α and IL-8 from THP-1 cells (Fig. 6G, H).

#### Inflammatory activity of CeN-treated LPS from *P. aeruginosa*

The LPS of *P. aeruginosa* when treated with CeN was also less stimulatory to THP-1 cells: compared to the untreated LPS, LPS treated with 0.1 mM CeN elicited significantly less TNF-α and IL-8 released from THP-1 cells (Fig. 6I, J). Treatment of THP-1 cells with CeN alone or PBS did not elicit any inflammatory response (Fig. 6I, J).

## DISCUSSION

Compared to SSD cream (*e.g.*, Silvadene^®^), CeN-SSD topical burn cream (*e.g.*, Flammacerium) produces a firm and dry eschar described as leather like or shell like, which is thought to resist bacterial colonization/infection, and is used, in addition to the general management of burn wounds, to facilitate postponement of eschar excision and autografting. To mimic CeN’ treatment of wound tissue and investigate its physical effects, we used two *in vitro* skin models: 3D-collagen matrix and *ex-vivo*–burned porcine skin. These models were treated with CeN solutions for 30 min to mimic the 30-min bathing in 40 mM CeN solution of severely burned patients that were associated with reduced mortality.^36^

We also studied CeN treatment of *P. aeruginosa* for effects on viability and *in vitro* virulence. Although the *ex-vivo*–burned porcine skin model used in this study was adapted from the previously developed studies,^37–39^ the current study has expanded the model to understand the mechanical properties of CeN-treated skin models, as well as *P. aeruginosa* penetration, which together add some novelty to the model.

### CeN effects on mechanical properties of skin models

CeN increased the stiffness of both skin models. In addition, the *ex-vivo*–burned porcine skin treated with CeN was less penetrable by *P. aeruginosa*, and *P. aeruginosa* inoculated on this CeN-treated skin model was more susceptible to killing by a silver dressing. Penetration of *P. aeruginosa* into *ex-vivo*–burned porcine skin pretreated with CeN was absent for up to 2 days for the highly invasive *P. aeruginosa* strain 1244.^30^

Penetration of *P. aeruginosa* into 3D-collagen matrix was also inhibited, such that more bacteria were confined to the superficial layers of the matrix, which may explain why bacteria inoculated on *ex-vivo*–burned porcine skin were more effectively killed by the silver dressing. This interpretation may also explain our previous *in vivo* finding that silver dressings supplemented with CeN were more effective than dressings alone at inhibiting the regrowth of normal flora in rat full-thickness burn wounds.^40^

CeN may change the properties of the skin models by cross-linking collagen in the 3D-collagen matrix. Similarly, in *ex-vivo*–burned porcine skin, CeN may cross-link native and burn-denatured macromolecules. Cerium ions exist in solution as Ce^3+^ or Ce^4+^ ions and have an ionic radius like calcium but with greater charge that contributes to high-affinity binding to Ca^2+^-binding sites on biological molecules.^41^ In our preliminary experiments (not shown), monomeric G-actin (rabbit skeletal muscle), high mobility group box-1 (HMGB1), and rat tail collagen were aggregated by CeN. Thus, cross-linking of macromolecules within burned skin may contribute to the observed changes in elasticity and tensile strength and the resistance to *P. aeruginosa* penetration.

An alternate possibility is that cerium ions available in the collagen gel and porcine skin reduce *P. aeruginosa* viability and motility. However, *P. aeruginosa* recovered from *ex-vivo*–burned porcine skin treated with or without CeN had similar viable counts and swarming motility. In addition, there was no detectable free cerium ions in the supernatants extracted from the CeN-treated *ex-vivo*–burned porcine skin (not shown). These results suggest that CeN in the skin models was not freely available at sufficient concentrations to affect viability or swarming motility of *P. aeruginosa* and that the action of CeN on the skin models was likely responsible for impeding *P. aeruginosa* penetration.

The burn eschar—dead tissue—produces toxins, including denatured macromolecules, which contribute to inflammation (local and systemic), immunosuppression, hypermetabolism, and at the extreme, multiorgan dysfunction.^42–44^ Due to the loss of skin barrier function, eschar is also a nidus for microbial infections that aggravate burn-induced inflammation.^17,43–45^ The findings that CeN stiffens *ex-vivo*–burned porcine skin may have some clinical implications. Increased stiffness may hamper bacterial penetration and delay the pathological progression of infection. In addition, stiffer skin, as a result of CeN ions cross-linking with the denatured macromolecules within the burned skin, some of which could be pro-inflammatory molecules such as damage-associated molecular patterns (DAMPs), may decrease systemic dissemination of these molecules and the resultant inflammation, as well as wound progression.^26,46–48^

### CeN effects on *P. aeruginosa* viability and virulence

Treatment of *P. aeruginosa* with CeN at 10–40 mM concentrations reduced their survival by ∼1.5–4 logs. The antimicrobial activity of CeN against gram-negative bacteria, including *P. aeruginosa*, likely results from positively charged cerium ions (Ce^3+^ and Ce^4+^) binding to negatively charged LPS. In an earlier study, CeN at ∼2 mM in saline (24 h treatment in suspension without shaking) caused *Escherichia coli* flocculation, outer membrane separation, and a nearly 1-log reduction in viable counts, suggesting that cerium ions interacting with LPS permeabilize the outer membrane.^49^ In addition, a CeN concentration as low as ∼0.02 mM produced membrane perturbations (ruffles) in *E. coli.*^49^

In our study, at sublethal 1 mM concentration, CeN reduced the swarming and twitching motility and other virulence properties of *P. aeruginosa*. Such an effect of CeN on LPS and outer membrane in *E. coli*^49^ may also explain the reduced swarming and twitching motility of *P. aeruginosa* as observed in our study. Alternatively, the reduction in swarming and twitching motility may be due to cerium ions binding to flagellar and pili proteins and interfering with their motor function. Reductions in *P. aeruginosa* swarming ability by specific mutations or treatment with a short cationic peptide (peptide 1018) in a murine model of skin abscess have been shown to reduce colonization and dissemination.^50^ The reduced swarming and twitching motility of *P. aeruginosa* after CeN treatment may contribute to the reduced penetration of *P. aeruginosa* through the skin models seen in this study.

Furthermore, CeN treatment of *P. aeruginosa* reduced their stimulation of the monocyte cell line THP-1 to produce inflammatory cytokines (TNF-α and IL-8). To explore why CeN-treated *P. aeruginosa* was less stimulatory to THP-1 cells, we tested LPS from *P. aeruginosa*. Treatment of *P. aeruginosa* LPS with CeN reduced its stimulation of cytokine production by THP-1 cells. Finally, CeN treatment of *P. aeruginosa* increased their phagocytosis by THP-1 cells. After CeN treatment, live and heat-killed *P. aeruginosa* was significantly more susceptible to phagocytosis. Similar to above, a possible explanation for these observations (reduced inflammatory stimulation and increased phagocytosis) is that Ce^3+^ or Ce^4+^ ions neutralize negative charges on LPS. Treatment of LPS with other positively charged molecules has shown to block the endotoxic effects of LPS.^51^

### Limitations

The *in vitro* and *ex vivo* models used herein are artificial systems that do not completely recapitulate the complex burn wound environment that includes systemic circulation, immune functions, and healing. Our studies also do not address the hypothesis that cerium ions can replace Ca^++^ from proteins, altering their structures and/or activities,^20^ the hypothesis that cerium ions bind tissue pyrophosphate, releasing calcium and pyrophosphate's inhibition on the formation of calcium phosphate crystals, as within cancellous or cortical bone,^52,53^ or the hypothesis that positively charged cerium ions interact with negatively charged bacterial membranes resulting in structural changes and bacterial growth inhibition. These mechanistic hypotheses warrant further exploration using *in vitro* and *ex vivo* models.

### Summary

Our data suggest multiple ways that CeN may reduce colonization and infection by acting on both the bacteria and the burn eschar: increasing phagocytosis of bacteria, decreasing pro-inflammatory cytokine production (possibly inhibiting wound progression), inhibiting swarming and twitching motility, and increasing the resistance of burn tissue to bacterial penetration. By inhibiting penetration, CeN treatment may confine *P. aeruginosa* to the superficial skin where vulnerability to killing by silver dressings may be increased. In addition, our studies demonstrate that *ex vivo* skin models may be an effective platform for screening treatments that interfere with bacterial penetration of wounded tissue.

## INNOVATION

Infection is the leading cause of mortality among burn patients and responsible for up to 50% of burn-related deaths. Multiple topical products have been developed to control burn infections to improve the healing. Conventional drug susceptibility testing focuses on identifying new drugs that exhibit bactericidal efficacy to a wide range of microorganisms. Our *in vitro* skin models offer a platform for screening drugs that change the property of burn tissues and result in preventing/retarding bacterial penetration/infection into wounded tissues.

**KEY FINDINGS**

- CeN treatment of *in vitro* skin models:
  o Increased stiffness of 3D collagen matrix and *ex-vivo*–burned porcine skin
  o Reduced penetration of *P. aeruginosa* into 3D collagen matrix
  o Increased vulnerability of *P. aeruginosa* inoculated on *ex-vivo*–burned porcine skin to killing by silver dressing.
- CeN treatment of *P. aeruginosa*:
  o Reduced viability
  o Reduced swarming and twitching motility
  o Reduced ability to penetrate 3D-collagen matrix and *ex-vivo*–burned porcine skin
  o Reduced stimulation of cytokine production by THP-1 cells
  o Increased vulnerability to phagocytosis by THP-1 cells.

## Supplementary Material

Supplemental data

Supplemental data

Supplemental data

Supplemental data

## ABOUT THE AUTHORS

**Shankar J. Evani, PhD,** is a Post-Doctoral Fellow at Combat Wound Care Group, United States Army Institute of Surgical Research, interested in wound healing and inflammation; host-drug-microbial interactions and drug discovery. **Ping Chen, PhD,** is Scientist at Combat Wound Care Group, United States Army Institute of Surgical Research. His research interests are microbiology, host-drug-microbial interactions in wound healing. **S.L. Rajasekhar Karna, PhD,** is Senior Research Scientist at The Geneva Foundation, Combat Wound Care Group, United States Army Institute of Surgical Research. His research interests are host-microbial interactions, wound healing, and drug discovery**. Peter D'Arpa, PhD,** is an investigator with The Geneva Foundation, a nonprofit organization that supports and advances medical research and education within the U.S. military.

His research expertise focuses on improving health within the U.S Military. **Kai P. Leung, PhD,** is a Capability Area Manager for Severe Burns and the science lead for the Combat Wound Care Group, U.S. Army Institute of Surgical Research, Fort Sam Houston TX. His research focuses on burn wound infection and repair, with ultimate goal of developing therapies for the wounded warrior.

## Abbreviations and Acronyms

ANOVA: analysis of variance
CeN: cerium nitrate
CFU: colony-forming unit
ctrl: control
ELISA: enzyme-linked immunosorbent assay
HBSS: Hank's balanced salt solution
IL-8: interleukin 8
LPS: lipopolysaccharide
MFI: mean fluorescence intensity
MRDC: Medical Research and Development Command
MRSA: Methicillin-resistant *Staphylococcus aureus*
PBS: phosphate-buffered saline
RPM: revolutions per minute
SD: standard deviation
SSD: silver sulfadiazine
TNF-α: tumor necrosis factor-α
