# Supplementary material for: Cerium Nitrate Stiffens In Vitro Skin Models and Reduces Pseudomonas aeruginosa Pathogenicity and Penetration Through Skin Models
Source: Adv Wound Care (New Rochelle). 2023 Jul 27;12(10):546–59. doi: 10.1089/wound.2022.0026 (PMC10387153; doi:10.1089/wound.2022.0026)
Supplement: Supplemental data [file Supp_FigS1.docx]

**Figure S1. CeN-treated porcine skin exhibited leather-like properties:** Porcine skins were cut into dumbbell shape and burned for 13 seconds at 100^o^C, treated with 0 (ctrl) or 40 mM cerium nitrate (CeN) for 30 minutes, and incubated at 37°C for 48 hours. After incubation, images were taken and representative images are shown.
