# Supplementary material for: Cerium Nitrate Stiffens In Vitro Skin Models and Reduces Pseudomonas aeruginosa Pathogenicity and Penetration Through Skin Models
Source: Adv Wound Care (New Rochelle). 2023 Jul 27;12(10):546–59. doi: 10.1089/wound.2022.0026 (PMC10387153; doi:10.1089/wound.2022.0026)
Supplement: Supplemental data [file Supp_FigS2.docx]

**Figure. S2**: **CeN or CeN+SSD treated burned porcine skins show no significant difference in skin stiffness.** Burned porcine skins either treated with CeN (40 mM) or CeN (40 mM)+ SSD (30 mM) were measured for Uniaxial tensile modulus/stiffness as described in Materials and Methods section 3.4. Data points represent mean ± SD from 2 experiments done in duplicates. ns, no significant difference (t-test) between groups.
