# Supplementary material for: Cerium Nitrate Stiffens In Vitro Skin Models and Reduces Pseudomonas aeruginosa Pathogenicity and Penetration Through Skin Models
Source: Adv Wound Care (New Rochelle). 2023 Jul 27;12(10):546–59. doi: 10.1089/wound.2022.0026 (PMC10387153; doi:10.1089/wound.2022.0026)
Supplement: Supplemental data [file Supp_FigS3.docx]

**Figure S3. CeN antimicrobial efficacy:** (A) Various bacteria as indicated in the graphs *were* incubated with 0 to 100 mM CeN for 24 h in saline. Colony-forming unit (CFU) counts were measured from the samples using TSA-blood agar plates and the data was graphed. Data points in the graphs represent mean ± SD obtained from 3 experiments done in duplicate. * Significant difference (*p*-value ≤ 0.05; One-way ANOVA) between control (ctrl; 0 CeN) and corresponding treatment groups.
