# Supplementary material for: Cerium Nitrate Stiffens In Vitro Skin Models and Reduces Pseudomonas aeruginosa Pathogenicity and Penetration Through Skin Models
Source: Adv Wound Care (New Rochelle). 2023 Jul 27;12(10):546–59. doi: 10.1089/wound.2022.0026 (PMC10387153; doi:10.1089/wound.2022.0026)
Supplement: Supplemental data [file Supp_FigS4.docx]

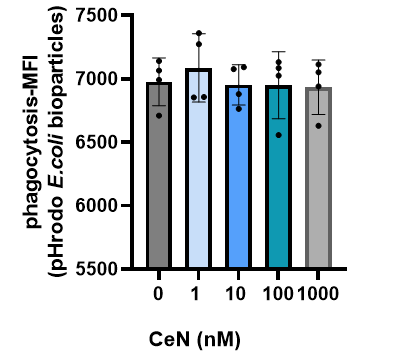


**Figure. S4**: **THP-1 cells treated with CeN showed no significant changes in phagocytosis.**

THP-1 cells (~ 1 × 10^6^/ml) were incubated with CeN (0, 1, 10, 100, and 1000 nM) overnight (~18h) in 5 ml polypropylene round-bottom tubes (Falcon Ref 352063, Corning Sciences, Mexico). Cells were stained with Incucyte Cytotox-green stain (catalog: 4633, Essen Bio science Inc, Ann Arbor, Michigan) and were then centrifuged at 150× g for 5 min, washed, and then presented to the pHrodo *E.coli* bioparticles (catalog: 4615, Essen Bio science Inc, Ann Arbor, Michigan) (5:1 ratio of normalized *E. coli* bioparticles to THP-1 cells in a total volume of 500 µl) for 2 hr at 37°C with 5% CO_2._ The pHrodo™ dye-conjugated bioparticles are non-fluorescent outside the cells but fluoresce brightly red in phagosomes. Phagocytosis was measured as the mean florescence intensity (MFI) of the phagocytized *E. coli* bioparticles using the Attune NxT flow cytometer (Invitrogen-Life Technologies, Carlsbad, CA USA). Data points are the mean ± SD from 2 experiments done in duplicate. No significance difference was observed.
